# Social support, depression, and heart disease: a ten year literature review

**DOI:** 10.3389/fpsyg.2013.00384

**Published:** 2013-07-01

**Authors:** Angelo Compare, Cristina Zarbo, Gian Mauro Manzoni, Gianluca Castelnuovo, Elena Baldassari, Alberto Bonardi, Edward Callus, Claudia Romagnoni

**Affiliations:** ^1^Department of Human and Social Sciences, University of BergamoBergamo, Italy; ^2^Psychology Research Laboratory, Istituto Auxologico Italiano IRCCSOspedale San Giuseppe, Verbania, Italy; ^3^Department of Psychology, Catholic University of MilanMilan, Italy; ^4^Pediatric and Adult Congenital Heart Disease Centre, IRCCS Policlinico San DonatoMilano, Italy; ^5^Cardiovascular Division, “L. Sacco” University General Hospital, University of MilanMilano, Italy

**Keywords:** depression, cardiac disease, social support, marital status, social relationship

## Abstract

**Background:** Coronary heart disease is the major cause of morbidity and mortality in the world. Psychosocial factors such as depression and low social support are established risk factors for poor prognosis in patients with heart disease. However, little is known about the hypothetical relationship pattern between them.

**Purpose:** The purposes of this narrative review are (1) to appraise the 2002–2012 empirical evidence about the multivariate relationship between depression, social support and health outcomes in patients with heart disease; (2) to evaluate the methodological quality of included studies.

**Method:** PubMed and PsychINFO were searched for quantitative studies assessing the multiple effects of low social support and depression on prognosis outcomes in patients with heart disease. The following search terms were used: social relation^*^, cardiac disease, support quality, relationship, and relational support.

**Results:** Five studies (three prospective cohort studies, one case-control study, and one randomization controlled trial) were selected and coded according to the types of support (social and marital). The majority of findings suggests that low social support/being unmarried and depression are independent risk factors for poor cardiac prognosis. However, all analyzed studies have some limitations. The majority of them did not focus on the quality of marital or social relationships, but assessed only the presence of marital status or social relationship. Moreover, some of them present methodological limitations.

**Conclusion:** Depressive symptoms and the absence of social or marital support are significant risk factors for poor prognosis in cardiac patients and some evidence supports their independence in predicting adverse outcomes. Cardiac rehabilitation and prevention programs should thus include not only the assessment and treatment of depression but also a specific component on the family and social contexts of patients.

## Introduction

The World Health Organization (2002) reports that coronary heart disease causes approximately 7.2 million deaths every year. There is increasing evidence that psychosocial factors are associated with Coronary Artery Disease (CAD) morbidity and poor prognosis in acquired heart disease conditions (Claesson et al., 2003; Rozanski et al., 2005, 1999; Aldana et al., 2006; Fukuoka et al., 2007; Compare et al., 2011a). Literature has outlined that depression is a primary risk factor for adverse outcomes in several cardiac populations. Both direct (biological) and indirect (behavioral) mediating processes explain the negative effect of depression on cardiac disease. The biological mechanisms include inflammatory and immune processes, alterations in activating HPA, variability in heart rate, increased activity of the sympathoadrenal and pituitary–adrenal axes, reduction in circulating endothelial progenitor cells, increase of cortisol and catecholamine levels, alteration of activities of autonomic nervous system and oxidation processes (Maier et al., 2007; Compare et al., 2011a,b, 2013; Nemeroff and Goldschmidt-Clermont, 2012). Moreover, unhealthy lifestyles often linked to depression, such as no medical adherence, increased consumption of tobacco, alcohol, and illicit substances, reduced physical activity, and overeating may contribute to a worse prognosis of cardiac disease as well (Maier et al., 2007). A further psychosocial risk factor for cardiac disease morbidity and mortality is low or no social support (Case et al., 1992; Williams et al., 1992; Hemingway and Marmot, 1999; Kuper et al., 2002). The association between social support and depression is particularly relevant because low social support may lead to the development or worsening of depression (Lett et al., 2005), while high levels of social support were shown to protect cardiac patients from the negative prognostic consequences of depression (Frasure-Smith et al., 2000). Although a plethora of studies have assessed the role of depression and low social support in the prognosis of cardiac patients, very few studies have evaluated the relationship pattern between them. The purposes of this narrative review are (1) to appraise the empirical evidence about the multivariate relationship between depression, social support, and prognosis outcomes in patients with heart disease; (2) to evaluate the methodological quality of studies.

## Literature search and study selection

**Figure 1:**
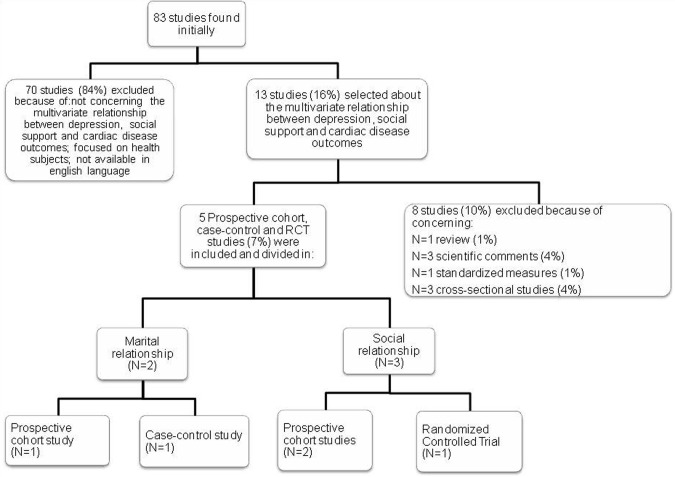
**Review Flow Chart**.

A search for English language articles that were published between 2002 and 2012 was performed in PubMed and PsychINFO using the terms social relation^*^, cardiac disease, support quality, relationship, and relational support, alone and in combination. In particular, target articles were searched using the following associations: ab(social relation^*^) AND ab(cardiac disease), ab(relation^*^ support) AND ab(cardiac disease), ab(support quality) AND ab(cardiac disease), ab(cardiac disease) AND ab(relationship), ab(cardiac disease) AND ab(relational support). After screening abstracts and removing reviews (1%), comments (4%), studies about standardized measures (1%), cross-sectional studies (4%), studies on healthy subjects and studies not concerning the multivariate relationship between depression, social support, and cardiac disease outcomes (84%), full-texts of remaining papers were retrieved and screened for final inclusion. No further paper was discarded and five studies (three prospective cohort studies, one control-case study, and one randomization controlled trial) were ultimately included in the narrative review (see Figure 1).

## Analysis

Main results and study flaws were summarized in a descriptive and narrative manner. Narrative review uses subjective, rather than statistical, methods to follow for reviews where meta-analysis is either not feasible or not sensible (Clarke and Oxman, 2003). Specific findings on the multivariate relationship between social support/marital status, depression and cardiac disease outcomes were mainly considered.

## Results

Five studies were identified and classified into two main groups: marital relationships and social relationships (see Table 1). In particular, two studies assessed the effect of marital relationships (one prospective cohort study and one case-control study), while three studies assessed the effect of social relationships (two prospective cohort studies and one Randomized Controlled Trial).

**Table 1:** **Examined studies concerning the multivariate relationship between depression, social support, and cardiac disease outcomes**.

**Study**	**Cardiac pathology**	**Methodology**	**Aim**	**Instruments**	**Results**
**Marital relationship (*N* = 2)**					
Chung et al., 2009	CHF	Prospective cohort study	To determine the effect of marital status on event-free survival in patients with heart failure who did or did not have depressive symptoms.	Beck depression inventory-II (BDI-II), interviews.	Marital status is an independent predictor of event-free survival, even after control for depressive symptoms and other covariates.
Empana et al., 2008	SCA	Case-control study	To investigate the risk of SCA associated with marital status, after accounting for other risk factors. To examined whether this association might be mediated by clinical depression.	Interviews.	Marital status and clinical depression both contributed to SCA risk, and clinical depression did not modify the risk associated with being unmarried.
**Social relationship (*N* = 3)**					
Lett et al., 2007	AMI	Randomized controlled trial	To compare the impact of network support and different types of perceived functional support on all-cause mortality or nonfatal reinfarction for patients with a recent acute myocardial infarction (AMI).	Diagnostic and interview schedule (DIS), hamilton rating scale for depression (HAMD), ENRICHD social support inventory, the interpersonal support and evaluation list, tangible support sub scale, the perceived social support scale, the social networks questionnaire.	High levels of depression are associated with increased risk of mortality and morbidity regardless of the level of support. Perceived tangible support and network support are not associated with better clinical outcomes regardless of level of depression.
Shen et al., 2004	CHD	Prospective cohort study	To identify the psychosocial factors that would independently predict patients' post-rehabilitation physical functioning while controlling for baseline status, risk stratification, age, and other psychosocial variables.	Quality of life after myocardial infarction (QLMI) scale, the brief COPE inventory, cook-medley hostility (Ho) scale, life orientation test (LOT), medical outcome study (MOS) social support survey, beck depression inventory (BDI).	Optimism and social support were found to contribute to health outcomes not only directly but also indirectly through the mediation of less engagement in detrimental coping and lower depressive symptoms.
Thurston and Kubzansky, 2009	CHD	Prospective cohort study	To examine associations between loneliness and risk of incident CHD over a 19-year period.	Center for epidemiologic studies of depression scale (CESD), interviews.	Loneliness was associated with incident CHD events, persisting with adjustment for demographic, behavioral, and biological risk factors as well as for depressive symptoms and the number of close friends and relatives.

AMI, acute myocardial infarction; CHD, coronary heart disease; CHF, congestive heart failure; SCA, sudden cardiac arrest.

### Marital relationships

Results about the key role that marital status has in predicting cardiac disease outcomes were similar between the two studies. Empana et al. (2008) used a case-control study to investigate the association between the risk of Sudden Cardiac Arrest (SCA) and marital status, exploring the possible mediation of clinical depression. The study sample consisted of 2119 cases (66.5 mean age; 71.2% male sex) and 4042 control (66.4 mean age; 69.4% male sex) participants that have experienced SCA. Controls were a stratified random sample of Group Health Cooperative (GHC) enrollees and had the same exclusion criteria as the cases. Cases were more likely than controls to have CHD risk factors and heart disease, a manual occupation, to be clinically depressed and less likely to be educated. Cases were more likely to be unmarried than the controls (30.2% vs. 21.0%), defined as being separated or divorced (10.0% vs. 7.0%), single (5.0% vs. 3.6%), or widowed (15.2% vs. 10.4%). The marital status at the time of SCA or a comparable index date for controls was assessed using ambulatory care medical records. Participants were classified as having depression if a physician reported the diagnosis of depression in the medical record within the year of the index date or if they were being treated with antidepressant medication. This study found that both marital status and clinical depression contributed to SCA risk in bivariate analyses, but only being unmarried, rather than depression, was significantly associated with an increased risk of SCA after taking into account other risk factors and clinical depression. Although being unmarried was associated to higher depression levels, authors have shown that risk of SCA associated with marital status was independent of clinical depression, probably due to the fact that unmarried subjects were less likely to receive preventive care, to engage in recommended healthy activities (medication compliance, exercise) and/or to obtain prompt assistance in case of symptoms of Coronary Heart Disease (Empana et al., 2008). Unfortunately, authors have not collected data about the perceived quality of the relationship between partners and other social connections, including friends and family members.

In the other study by Chung et al. (2009), the effects of marital status on event-free survival in patients with heart failure who did or did not have depressive symptoms were explored. Patients with heart failure were followed-up for up to 4 years after assessment of depressive symptoms. Data on death and hospitalization were collected. Depressive symptoms were assessed by using the Beck Depression Inventory-II (BDI-II), a valid and reliable instrument used to predict mortality and hospitalizations in CHD patients and patients with heart failure. Marital status was assessed via patient interviews. Data on marital status were initially coded in five categories: married, single, widowed, divorced, and cohabitated. This variable was then dichotomized into married and non-married. The unmarried group consisted of single, widowed and divorced patients. The married group included married and cohabitating patients. Patients' demographic (sex, age, ethnicity, marital status, education, income) and clinical [left ventricular ejection fraction, medications, comorbid conditions, New York Heart Association (NYHA) class] characteristics were collected by reviewing medical charts with a structured questionnaire. After data collection, patients were grouped into those with (*n* = 55; 57.8 mean age; 67% male sex) and those without (*n* = 111; 62.4 mean age; 69% male sex) depressive symptoms and into groups of married (*n* = 93; 61.1 mean age; 82% male sex) and non-married (*n* = 73; 60.6 mean age; 52% male sex). Marital status was found to be an independent predictor of event-free survival, even after controlling for depressive symptoms and other covariates (Chung et al., 2009). However, this study is limited because it did not investigate the quality of the marital relationship.

### Social relationships

Shen et al. (2004) studied the psychosocial factors that might independently predict patients' post-rehabilitation physical functioning, while controlling for baseline status, risk stratification, age, and other psychosocial variables such as social support and depression. The sample was comprised of 138 mens and 4 womens with a mean age of 62.15 years old (*SD* = 9.68) referred to a cardiac rehabilitation phase II program, a comprehensive program offering exercise, health education, and psychosocial interventions. Prior to intervention, participants completed a battery of health and psychosocial questionnaires. The baseline and post treatment physical functioning was assessed by the Quality of Life After Myocardial Infarction (QLMI) scale, a valid, and reliable instrument to measure several specific areas of functioning, including cardiac symptoms, physical restrictions, confidence, and self-esteem (Hillers et al., 1994). The 18-item Medical Outcome Study (MOS) social support survey was used to assess positive interactions and several functional support areas received by the individual, including emotional, informational, tangible and affectionate support (Espnes, 1995). The BDI (Beck et al., 1961) was employed to assess the severity of the patient's depressive symptoms. Patient's severity of illness at entry and risk for future events were assessed by a composite index score largely based on the recommended risk stratification guidelines (Roitman et al., 1998). Also optimism, coping style and hostility were assessed by standardized instruments (Scheier and Carver, 1985; Barefoot et al., 1989; Carver, 1997). They found that optimism and social support contributed to health outcomes not only directly but also indirectly through the mediation of less engagement in detrimental coping and lower depressive symptoms (Shen et al., 2004). However, a limitation in this study concerns the imbalanced nature of the sample (138 mens vs. 4 womens); women were few in the study, thus limiting the generalizability of results to female patients.

Thurston et al.'s prospective cohort study (2009) focused on the association between loneliness and risk of incident CHD in a sample of women over a 19-years period. Hypotheses were examined using data from the First National Health and Nutrition Survey (NHANES I), a multistage, national probability survey conducted between 1971 and 1975, and its follow-up studies. Participants were asked to rate the statement: “I feel lonely” on a 4-point scale from “rarely or none of the time” to “most of the time” in the past week. Due to small cell sizes and the categorical nature of this measure, loneliness scores were categorized as low (*n* = 1966; mean age 45.3; *n* = 960 male), medium (*n* =409; mean age 43.0; *n* = 128 males), and high (*n* = 241; mean age 44.0; *n* = 62 males) for primary analyses. CHD events were identified via hospital/nursing home discharge reports and death certificates. Follow-up interview included the question, “In general, how many relatives and friends do you have that you feel close to? These are people whom you feel at ease, can talk to about private matters, and can call on for help.” This question was used as a proxy measure of social networks to ensure that effects of loneliness are not simply reiterating the well-known relationship between social networks and disease. Findings showed that loneliness predicted incidence of CHD events, even after adjusting for demographic, behavioral and biological risk factors as well as for depressive symptoms and number of close friends and relatives (Thurston and Kubzansky, 2009). However, the study's findings should be interpreted in light of several limitations such as the single-item assessment of loneliness.

Finally, in Lett et al.'s study (2007) the impact of network support and different types of perceived functional support on all-cause mortality or nonfatal re-infarction were assessed in patients with a recent acute myocardial infarction (AMI). Participants were recruited from the Enhancing Recovery in Coronary Heart Disease (ENRICHD) trial; 2481 AMI patients with depression or low social support were randomized to a cognitive–behavioral intervention or to a usual care control group. The average age of the sample was 61 years; 44% of the sample was female and 56% was male. In ENRICHD, patients with a recent AMI were screened for depression or low perceived social support. Four measures of social support were used: The Interpersonal Support and Evaluation List, Tangible Support subscale (Cohen et al., 1985), that is a 10-item scale designed to assess the perceived availability of tangible aid; the Perceived Social Support Scale (Blumenthal et al., 1987), that is a 12-item scale that measure support received from friends, family members, and significant others; the Social Networks Questionnaire (Glass et al., 1997), that measures the proximity, size, frequency of non-visual and visual contacts and reciprocity in four domains: children, one special confidant, friends and acquaintances; the ESSI is a 7-item measure developed as a screening tool for the ENRICHD study to measure perceived emotional support, instrumental support, appraisal support, and marital status probes. Depression was assessed with the Diagnostic and Interview Schedule (DIS) combined with the Hamilton Rating Scale for Depression (HAMD), yielding a *Diagnostic and Statistical Manual of Mental Disorders* (American Psychiatric Association, 1994) diagnosis and severity rating of depressive symptoms. High levels of depression were shown to be associated with increased risk of mortality and morbidity, regardless of the level of social support. Perceived tangible support and network support were not associated with better clinical outcomes regardless of the level of depression. This means that high levels of perceived social support did not eliminate the increased risk of depression on clinical events. Furthermore, Lett's study showed that the patients' subjective appraisal of the unavailability of or dissatisfaction with support appears to be a more important psychosocial risk factor for CHD than a limited social network (Lett et al., 2007). In this study, the lack of a non-depressed and non-socially isolated control group is the major limitation.

## Discussion

In this review, studies that investigated the multivariate relationship between depression, social support (marital and social) and cardiac disease outcomes were described. Although findings are mixed, the majority of them (see Table 2) suggests that low social support/being unmarried and depression are independent risk factors for poor cardiac prognosis (Lett et al., 2007; Empana et al., 2008; Chung et al., 2009; Thurston and Kubzansky, 2009) and does not support the multivariate hypothesis. Actually, some studies have outlined that depression does not moderate the significant risk related to being unmarried (Empana et al., 2008; Chung et al., 2009). On the other hand, Lett et al.'s study (2007) focused on the positive effect of social support and found that a high level of social support does not moderate the negative effects of depression. Thurston and Kubzansky (2009), instead, outlined the key role of loneliness, rather than depression, defined as an aversive emotional response to a perceived discrepancy between desired and actual level of social interaction (Hawkley et al., 2003). Thurston's study results (2009) show that perceived loneliness had a role in influencing cardiac disease independently of depression or number of social interactions. Only one study (Lett et al., 2007) focused on the high level of social support and its possible positive effects on depression symptoms. This highlights a bias in literature because the majority of studies has analyzed the negative effects of a low social/marital support or their absence, rather than the positive effects. However, all analyzed studies have some limitations. The majority of them did not measure the quality of marital or social relationships, but assesses only the presence of marital status or social relationship (Lett et al., 2007; Empana et al., 2008; Chung et al., 2009); some of them present methodological limitations, such as the lack of adequate control groups (Lett et al., 2007), a high gender imbalance (Shen et al., 2004) and the use of a single-item scale (Thurston and Kubzansky, 2009).

**Table 2:** **Topic aspects emerged by literature review**.

**Topic aspects emerged by literature review.**
There is an increasing evidence of the association between social support and cardiac disease, on one hand, and depression and cardiac disease on the other hand. The association between social support and depression is particularly relevant because depression itself is known to be an important outcome predictor for cardiac patients and, on the other hand, low social support levels are important risk factors for the subsequent development or worsening of depression. The majority of studies suggests that low social support/being unmarried and depression are independent risk factors for poor cardiac prognosis and does not support the multivariate hypothesis. The majority of studies did not focus on the quality of marital or social relationships, but assessed only the presence of marital status or social relationship. Moreover, some of them present methodological limitations. It would be desirable that future research and clinical protocols consider the psychological aspects of cardiac patients and the relational context of the patient as moderating variables. Identifying and reinforcing current social support networks and its quality is helpful to improve adherence behaviors and quality of life, in particular of patients with depressive symptoms.

In only one study (Shen et al., 2004) a mediating relationship between social support and depression was found. Actually, Shen et al.'s study (2004) shows that social support contributed to health outcomes and cardiac disease not only directly but also indirectly through the mediation of lower depressive symptoms (Shen et al., 2004). Previous literature showed that the quality of social or marital relationships is important in the link between depression and cardiac disease (Frasure-Smith et al., 1995, 2000). The health benefits of relationships are moderated by their affiliative quality and the subject's cognitive appraisal of supportive resources availability (Cohen and Wills, 1985; Coyne et al., 1990; Lyons et al., 1995). Studies postulated that high social support levels can buffer or modulate cardiovascular responses to acute psychological stressors (Thorsteinsson et al., 1998). In contrast, negative social interactions can heighten cardiovascular responses in laboratory settings (Robles and Kiecolt-Glaser, 2003).

In conclusion, very few quantitative studies concerning the multivariate relationship between social support, depression and heart disease outcomes were published from 2002 to 2012. Regarding the positive role of the quality of relationships, it would be desirable that future research and clinical protocols consider the psychological aspects of cardiac patients (Compare et al., 2012; Grossi et al., 2012) and the relational context of the patient as moderating variables.

## Clinical consideration

Depressive symptoms and social support may be taken into account by nurses, psychologists, and physicians to structure effective interventions, with the aim of improving health outcomes and reducing the risk of mortality and morbidity in cardiac patients. During the evaluation and waiting list for heart surgeries and interventions, relatives, and friends may help cardiac patients to cope with physical disability, limitation of personal autonomy and with a sense of uncertainty about the future. It was proposed in the literature to extend psychosocial interventions aimed at improving mental health, quality of life, and compliance after a cardiac event to family caregivers (Dew et al., 2004). Recently, intervention programs such as peer mentor support have been developed to improve or enhance social support in patients with heart failure (Daugherty et al., 2002; Riegel and Carlson, 2004; Villani et al., 2007; Manzoni et al., 2011). Identifying and reinforcing current social support networks and their quality is helpful to improve adherence behaviors, in particular in patients with depressive symptoms. Future reviews should include more studies to have a complete overview of the topics and to provide the basis to investigate how social support can be enhanced by nurses, families, and psychologists in patients at risk of recurrent cardiac events and high levels of depression.

### Conflict of interest statement

The authors declare that the research was conducted in the absence of any commercial or financial relationships that could be construed as a potential conflict of interest.
